# Physical fitness data monitoring of college students based on the internet of things and blockchain

**DOI:** 10.3389/fpubh.2022.940451

**Published:** 2022-09-09

**Authors:** Yunpeng Sang, Lijun Wang

**Affiliations:** ^1^Sport Department, Changshu Institute of Technology, Suzhou, China; ^2^Institute of Physical Education and Health, Yulin Normal University, Yulin, China

**Keywords:** physical fitness data monitoring, blockchain technology, internet of things, data collection, blockchain system

## Abstract

Contemporary college students are the pillars of the country and bear the responsibility of building a great country. College students should not only have smart brains, but also have strong bodies. The state has always attached great importance to the physical condition of college students and has promulgated a series of relevant policies and regulations to ensure the effective development of college students' physical health work. This paper aims to monitor and research college students' physical fitness data based on the Internet of Things and blockchain technology. This paper first introduces the data collection based on the Internet of Things, the Internet of Things data collection system has good versatility, ease of use, and quite rich functions, which can realize the collection and reliable transmission of different environmental data. Then focuses on the data collection and confidentiality technology based on blockchain. Each user in the blockchain system has a pair of public and private keys, and elliptic curve algorithms are usually used to generate public key cryptography. Finally, based on the Internet of Things and blockchain technology, the physical fitness data of college students is analyzed and researched. The experimental results of this paper show that, according to the data collection technology of the Internet of Things and blockchain, the analysis of variance is carried out on the data of male pull-ups and female sit-ups of 2019 students. The analysis of variance F of boys' pull-ups is 76.222, and the significance is about 0, that is, *P* < 0.01. The difference is very obvious, which proves that there is a significant difference in boys' pull-ups in the past 3 years. The analysis of variance F for girls' sit-ups is 89.187, and the significance is about 0. Similarly, it shows that there are significant differences in girls' sit-ups in the past 3 years. Therefore, the existing teaching mode is stabilized and physical exercise is enhanced. Meanwhile, to enhance the physical fitness of students, it is necessary to strengthen the strength of physical education teachers and increase the introduction of sports talents and business training.

## Introduction

Physical health is a key factor in the overall quality of the nation. The level of national physical health is not only an issue of personal health, but also a fundamental issue related to whether a nation can stand on its own in the forest of nations in the world. Students' physical work is also an important part of developing physical education and health education, and the evaluation of primary and secondary school students' health is also the main content of students' physical work. At present, governments all over the world attach great importance to the health status of young primary and secondary school students. Especially in some countries, the government tests the health status and major health problems of local young primary and secondary school students every year to understand the development and changes of students' health status, and to formulate relevant, providing relevant information on national cultural policies and regulations, sports system regulations, etc. However, the physical health of primary and secondary school students is not optimistic. In a long-term monitoring survey on the physical condition of primary and secondary school students across the country, it was found that the physical quality and function indexes of primary and secondary school students across the country such as speed, explosive power, athletic strength and vital capacity were greatly weakened, while the number of undernourished, obese and overweight primary and secondary school students increased significantly. Therefore, a monitoring research on college students' physical fitness data based on the Internet of Things and blockchain technology is conducted.

Timely monitoring and effective feedback on students' physical health are implemented to help students achieve physical health indicators. It arouses the enthusiasm of students to participate in sports spontaneously, helps students form a healthy lifestyle and reasonable behavior habits, and makes the campus and community pay more attention to students' mental health, thereby cultivating students' comprehensive quality. Meanwhile, this research also helps physical education teachers to propose differentiated teaching contents according to the physical conditions of different groups of students. It can also help teachers to carry out sports courses in a targeted manner, formulate sports courses, methods and measures, and carry out scientific curriculum research. This will have a positive effect on improving the quality and teaching effect of the school's sports. Meanwhile, this paper conducts research and discussion on the monitoring of college students' physical fitness data based on the Internet of Things and blockchain, to make a certain contribution to the physical health of college students and lay a certain foundation for future research in this area.

The innovation of this paper is reflected in: (1) The data acquisition system of the Internet of Things is expounded, and the structure diagram of the data acquisition system is given; (2)The data collection and confidentiality technology based on blockchain is introduced. Each user in the blockchain system has a pair of public and private keys, and the elliptic curve algorithm is usually used to generate public key cryptography; (3)Based on the Internet of Things and blockchain technology, the experimental detection and analysis of the physical fitness data of college students is carried out.

## Related work

According to the research progress in foreign countries, different researchers have also conducted corresponding cooperative research in the monitoring of institutional data of college students: Zakariya N gave a literature review of machine learning techniques. He categorized and structured the published research evidence in the field of machine learning techniques for predicting physical activity using fitness data based on personal background and fitness data to predict appropriate physical activity. This research provided new insights into software development in healthcare technology to support the personalization of individuals in managing their own health (1). Wang H conducted inquiries, modifications, additions, deletions, etc. to students, we and conducted big data analysis of useful information to enhance students' physical health information management capabilities. In addition, Wang H also established a doctor recommendation model based on online question and answer, and gave specific health advice for students with different physiques (2). Masanovic B aimed to focus on reviewing the literature on physical activity and physical health in children and adolescents to identify and assess current conditions and provide a better basis for creating future monitoring systems (3). However, these scholars lack a certain technical argument for the system data detection of college students. For this, some literatures based on blockchain have been consulted.

Some scholars also have some research on blockchain: From a security standpoint, Kshetri clarified how a blockchain solution can outperform most of the current Internet of Things natural ecosystems that rely on aggregated cloud servers in many ways. By comparing real-world applications with real-world examples, it is believed that the decentralized nature of blockchains is likely to cause malicious actors to be less susceptible to manipulation and forgery (4). Sharma P K provided a distributed SDN framework for Internet of Things using blockchain network (DistBlockNet). It followed the principles required to design a secure, scalable, and efficient network architecture. The DistBlockNet model of Internet of Things architecture combines the advantages of two emerging technologies: SDN and blockchain technology (5). Kshetri N evaluated the effect of blockchain technology in improving security and ensuring information security. Because a lot of data is currently stored in cloud data centers, it also compares the performance of the blockchain in comparison to the cloud in various aspects such as security and privacy (6). However, these scholars did not conduct research and discussion on the monitoring of college students' physical fitness data based on the Internet of Things and blockchain, but only discussed its significance unilaterally.

## Methods of college students' physical fitness data monitoring based on internet of things and blockchain

Both physical development and human social and mental health involve the physical development, physiological mechanisms, sports performance, social and mental health status, and community adaptation of the human body, which shows that there are both differences and close connections between them. The body is an important basic element of human life activities, and it is also the most important substance for physical health. Constitution is the study of the human body from the “outside,” and health is the study of the human body from the “inside.” The relationship between body and health refers to the relationship between the “quality” and “state” of an object, because all objects have quality, and the quality of the body is the physique. The body is the external manifestation of this quality of human beings – a state of mind. Physical function is the ability of human beings to maintain a healthy mental state. In this regard, this paper studies and discusses the monitoring of college students' physical fitness data based on the Internet of Things and blockchain, to make certain contributions to the physical health of college students.

### Internet of things data collection

The Internet of Things data acquisition system has good versatility and ease of use. Its functions are quite rich, and it can realize the collection and reliable transmission of different environmental data to meet the practical application in more scenarios, and it is convenient for staff related to other Internet of Things business to carry out secondary development. Therefore, the data acquisition system should meet the following design requirements: It can provide support to connect with various front-end systems and external devices; it can support long-distance and short-distance transmission, ensuring safe and stable transmission of data when collecting data; it can cope with the occurrence of abnormal conditions, thus ensuring safe storage; it has a good upper-level application management platform, which is convenient for users to access the collected data at any time (7).

**Figure 1:**
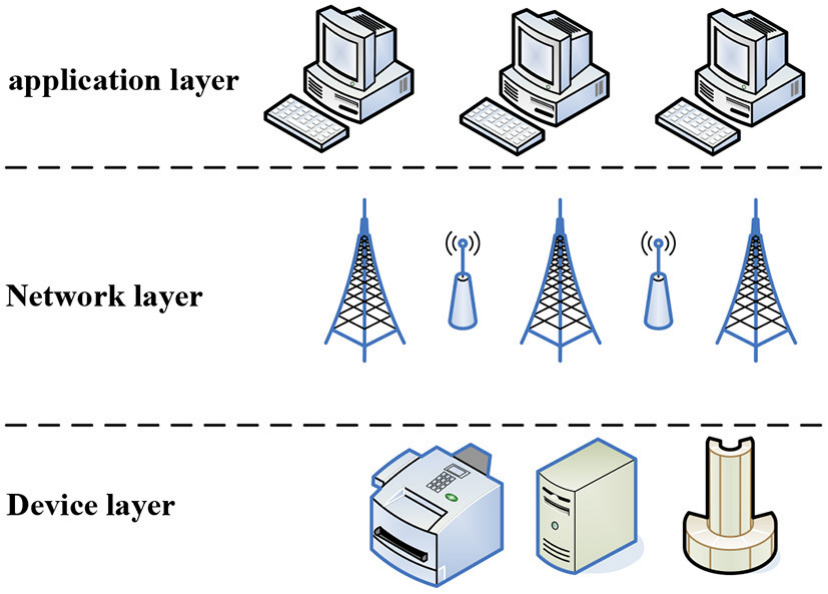
Data acquisition system structure.

The structure of the Internet of Things data acquisition system is shown in Figure 1. According to different application scenarios, different equipment terminals, networking methods, and upper-level operation management platforms are adopted. In the process, it can be divided into the facility layer, the network system layer, and the operation layer.

### Basic structure of blockchain

A blockchain consists of blocks and chains. Blocks are linked together in chronological order by the hash value of the previous block, which makes the blockchain difficult to change. If the value of a block needs to be changed, the block headers of all blocks behind the block also must be changed, otherwise the entire blockchain will be broken (8). Therefore, immutability is a key property of blockchains, which makes it impossible to modify any information without network consensus. Blocks are time-limited when writing data, which means that after a certain time, these blocks will become a permanent part of the blockchain and their data cannot be deleted or changed. For example, in Factorde and Bitcoin, a block is added to the chain every 10 min. In Ethereum, the time to generate a new block is shorter, and a new block can be generated every 15 s (9). Network participants process transactions in the blockchain by verifying the legitimacy of newly generated blocks and storing that transaction in the block. The success of a transaction can be verified by checking the signature of the transaction and checking the existence of a previous transaction in the same ledger. When the transaction size of a node in the running pool, that is, the set of transactions that have not been added to the blockchain, reaches a predefined size (block size), the node will generate a new block by executing the consensus algorithm (10).

Taking Bitcoin as an example, a block consists of a block header and a block body. The block header consists of an index, a timestamp, the hash value of the block, and the hash value of the previous block (prev-hash). The hash value is generated by a hash function, and the hash of each block in the blockchain is uniquely determined and cannot be changed (11). The hash value obtained by hashing any data is unique. If the input data is the same, the hash value is equal. Therefore, it is almost impossible to change the hash value by changing the input data (12). Due to this feature, hash values are used to verify the consistency of data. The block body stores the transaction information of the block, and the data in the block is stored in the Merkel tree in the block.

#### Hash algorithm

Hash algorithm is an essential technology in the field of information technology and is widely used in blockchain. After the binary data of any length is mapped by the hash function, it will be converted into a fixed-length output. The output value is the hash value of the data. There are many hash functions, and the most used in blockchain technology is the hash algorithm (13). Binary data of any length can be converted into a 256-bit 32-byte hash value by this algorithm. The hash algorithm also has the advantages of one-way, randomness, timing, and fixed length (14).

(1) One-way: The output data can be easily obtained from the input data, but the input data cannot be calculated from the output data.
(2) Randomness: Even if only a small change is made to the input data, the output data may change dramatically, and the impossible input data will generate the same hash value.
(3) Timing: Any input data of different lengths take approximately equal time through the same type of hash algorithm.
(4) Fixed length: It takes approximately the same amount of time to perform the same type of hash operation on input data of different length.

Hash functions are usually defined in terms of generating hash values. There are two main methods.

#### Hash algorithm based on addition and multiplication

A hash based on addition and multiplication is implemented by iterating over the elements in the data and then by incrementing each initial value associated with the data element. Usually the value of an element is multiplied by a prime number (15).

The calculation method of the hash based on addition and multiplication is given as Formula 1, Formula 2, Formula 3, and Formula 4:


(1) $$\begin{array}{l} l\left(s\right)=l^{-1}⊕\left(s⊗t\right) \end{array}$$

(2) $$\begin{array}{l} l\left(s\right)=\overset{\left|s\right|}{\underset{n=0}{\sum }}s_{n}⊗t_{n} \end{array}$$

(3) $$\begin{array}{l} l\left(s\right)=l^{-1}⊗\left(s⊗t\right) \end{array}$$

(4) $$\begin{array}{l} l\left(s\right)=\overset{\left|s\right|}{\underset{n=0}{\prod }}s_{n}⊗t_{n} \end{array}$$

#### Shift-based hash algorithm

Similar to additive hashing, shift-based hashing also utilizes each element in the string data. However, unlike additive hashing, shift hashing is more inclined to shift data (16). It is usually a combination of left and right shift operations, and the number of shifts is the quality. The result of each shift is just some more accumulation operations, and the final result of the shift calculation is the final result of the shift hash calculation. The formula for the shift hash algorithm is as Formula 5, Formula 6, and Formula 7:


(5) $$\begin{array}{l} l\left(s\right)=l^{-1}⊕\left(s⋘t\right)⊗\left(s⋙t\right) \end{array}$$

(6) $$\begin{array}{l} l\left(s\right)=\overset{\left|s\right|}{\underset{n=0}{\sum }}\left(s_{n}⋘t_{n}\right)⊗\left(s_{n}⋙t_{n}\right) \end{array}$$

(7) $$\begin{array}{l} l\left(s\right)=\overset{\left|s\right|}{\underset{n=0}{\prod }}\left(s_{n}⋘t_{n}\right)⊗\left(s_{n}⋙t_{n}\right) \end{array}$$

#### Structure of a Merkel tree

A Merkel tree is a hash binary tree, in which each leaf node is the hash value of a transaction. For example, in Bitcoin, Merkel trees are used to summarize all transactions in a block, while generating a digital fingerprint Merkel tree root that contains the entire transaction set. Generating a Merkel tree requires a recursive hash calculation every two nodes to generate a new hash value and store it in the Merkel tree until there is only one hash value at the end (17). The final hash, which is the Merkle root of all transactions in these blocks, is stored in the block header. As long as the number of leaf nodes is an odd number, an even number of leaf nodes can be generated by repeating each time a transaction is ended.

The calculation method of the Merkel tree is as follows, taking a Merkel tree with only four leaf nodes as an example.

1) Two SHA256 hash algorithms to hash each transaction data are used to calculate the hash value of each transaction. The four hash values of *L*_*W*_, *L*_*X*_, *L*_*Y*_, *L*_*Z*_ can be calculated, that is, the four leaf nodes of the Merkel tree (18). *L*_*W*_ is taken as an example:


(8) $$\begin{array}{l} {\text{L}}_{\text{W}}=\text{SHA}256\;\left(\text{SHA}256\;\left(\text{Deal A}\right)\right) \end{array}$$

2) The hash values of the two leaf nodes *L*_*W*_ and *L*_*X*_ are also combined with two SHA256 hash calculations to obtain a new hash value *L*_*WX*_. The same calculations on *L*_*Y*_ and *L*_*Z*_ are performed to get another hash value *L*_*YZ*_. The calculation of *L*_*WX*_ is taken as an example:


(9) $$\begin{array}{l} {\text{L}}_{\text{WX}}=\text{SHA}256\;\left(\text{SHA}256\;\left({\text{L}}_{\text{W}}+{\text{L}}_{\text{X}}\right)\right) \end{array}$$

3) The same combined hash calculations as the above steps with the existing two hash values *L*_*WX*_ and *L*_*YZ*_ are performed. Finally, a new hash value *L*_*WXYZ*_ can be obtained. At this point, there is no node with the same height as *L*_*WXYZ*_ in the Merkel tree, so *L*_*WXYZ*_ is the Merkel root of the Merkel tree. The 32-byte hash value of this node will be written into the Merkel root field of the block header, and the calculation process of the Merkel tree is completed (19). The calculation method of *L*_*WXYZ*_ is as Formula 10:


(10) $$\begin{array}{l} {\text{L}}_{\text{WXYZ}}=\text{SHA}256\;\left(\text{SHA}256\;\left({\text{L}}_{\text{WX}}+{\text{L}}_{\text{YZ}}\right)\right) \end{array}$$

#### Asymmetric encryption algorithm

Cryptographic calculations can be divided into symmetric encryption algorithms and asymmetric encryption algorithms. Asymmetric encryption algorithm is a cryptographic calculation commonly used in blockchain, and its security is much higher than that of symmetric encryption algorithm. In an asymmetric encryption algorithm, two asymmetric keys (public key and private key) are used separately in the encryption and decryption processes (20). It has two characteristics: First, after the data is encrypted by any one of the keys, only the key corresponding to the password used in the encryption process can be used to decrypt the data; the second is the public key password that can be stolen by everyone, while the private key is strictly kept secret.

**Figure 2:**
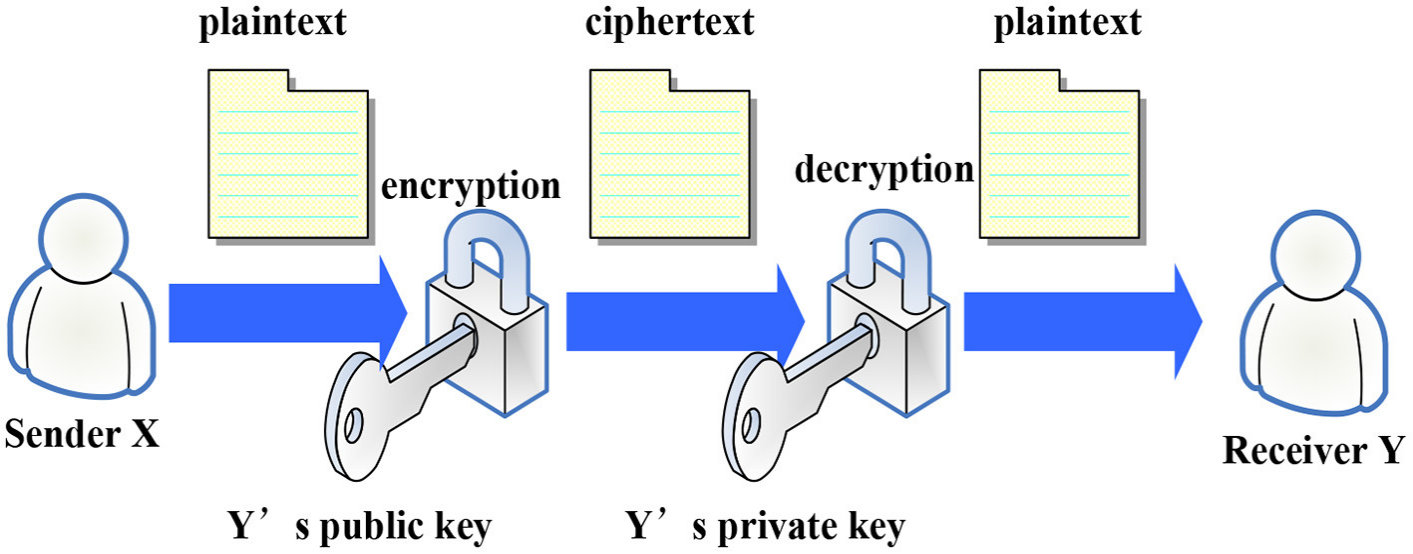
Encryption algorithm.

Asymmetric encryption algorithms are mainly used in blockchain to encrypt and sign data and for registration authentication. The basic calculation process of the encryption algorithm is shown in Figure 2. The sender X first encrypts the original message by giving the receiver Y's public key and obtains the ciphertext, and then passes the secret file to Y. After receiving the ciphertext, Y, deciphers the information through his private key, which is the algorithm used to encrypt the transaction information in Bitcoin.

**Figure 3:**
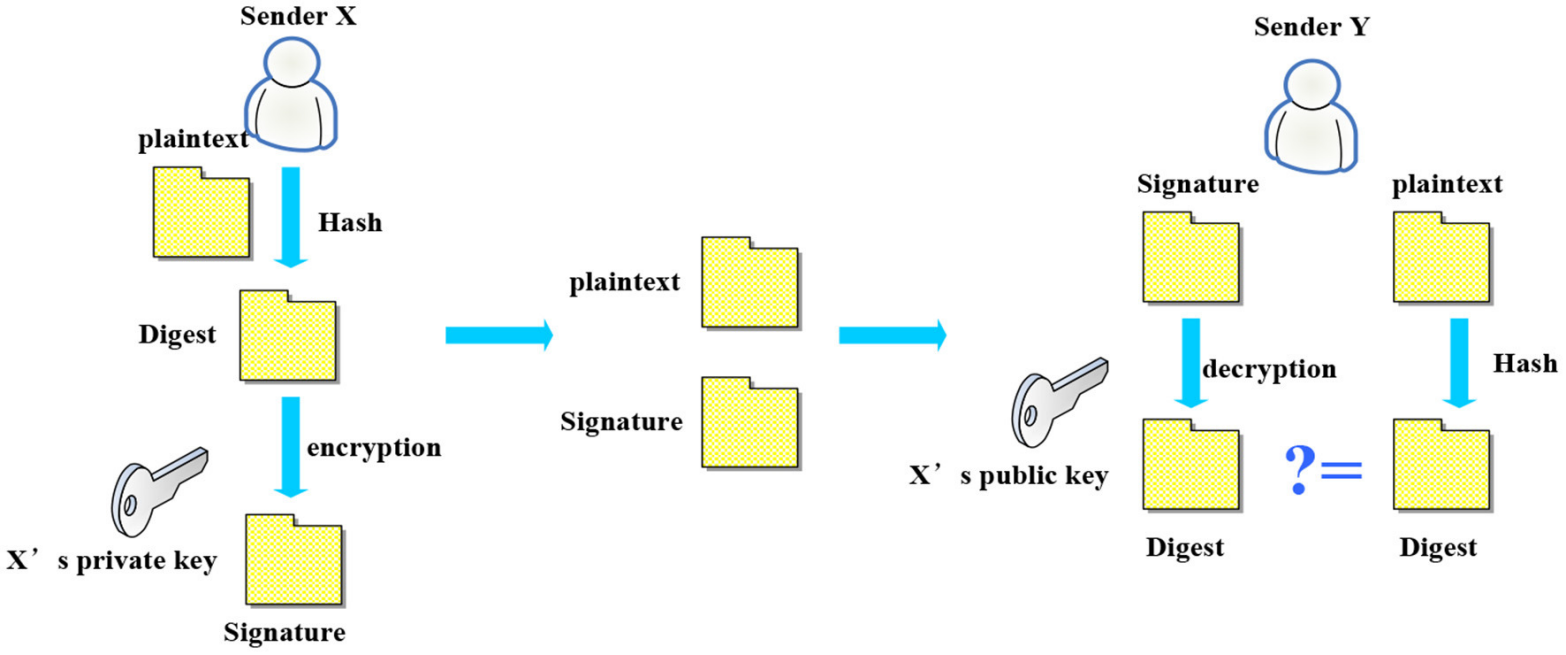
Digital signature algorithm.

The calculation process of the digital signature algorithm is shown in Figure 3. The original message is hashed by the sender X to generate a message digest, and then the data is encrypted and signed by its own private key to form a digital signature. Finally, the original data and digital signature are transmitted to the receiver Y in the form of blocks. After the signal is accepted by recipient Y, the digital signature is interpreted as message digest one using recipient X's public key, and the original message is hashed into another message digest two by the recipient. Finally, whether the two information digests are the same should be compared. If it is the same, the receiving facility accepts the original message and performs the corresponding action. If it is different, it will be discarded. Therefore, it can be guaranteed that the information is sent by the sender X, and has not been tampered with by others during the message transmission process.

The information traded on the blockchain can be seen by anyone, and the identity information of the account owner is also highly confidential, so each user can ensure their own information security by setting a variety of pseudonyms. Only those who have obtained the permission of the data owner have the right to access so that the data security and the user's privacy can be effectively guaranteed. Each user in the blockchain system has a pair of public and private keys, and elliptic curve algorithms are usually used to generate public key cryptography.

The basic concept of an elliptic curve refers to the set of all points of an elliptic curve on the projective plane that conform to the Weierstrass equations, and there is no singular point on the curve.


(11) $$\begin{array}{l} B^{2}C+s_{1}ABC+s_{3}BC^{2}=A^{3}+s_{2}A^{2}C+s_{4}AC^{2}+a_{6}C^{3} \end{array}$$

The projective plane coordinate system is an extension of the Cartesian coordinate system and adds the definition of an infinite point. In this coordinate system, if there is an intersection between two perpendicular curves that intersect each other, the intersection is an infinite point. A projective plane coordinate system can be converted to a Cartesian coordinate system by: Let s (a, b) be any point in the Cartesian coordinate system, and let:


(12) $$\begin{array}{l} \text{s}=\text{AC},\text{b}=\text{BC} \end{array}$$

The coordinates of point s in the projective plane coordinate system are *(A, B, C)*. For example, *(2, 3)* can be converted to *(2C, 3C, C)*.

The above formulas with the equation in the Cartesian coordinate system are converted as Formula 13:


(13) $$\begin{array}{l} {\text{b}}^{2}+{\text{s}}_{1}\text{ab}+{\text{s}}_{3}\text{b}={\text{a}}^{3}+{\text{s}}_{2}{\text{a}}^{2}+{\text{s}}_{4}\text{a}+{\text{s}}_{6} \end{array}$$

The definition of elliptic curve in cryptography is: Define an elliptic curve in the finite field *Gr*, and the expression of the curve is as Formula 14:


(14) $$\begin{array}{l} {\text{b}}^{2}={\text{a}}^{3}+\text{sa}+\text{t} \end{array}$$

The additional conditions are as follows:

1) There are only *r* prime elements in *Gr*.
2) In *Gr*,
  (15) $$\begin{array}{l} \text{s}+\text{t}\equiv \text{u}\left(\text{modr}\right) \end{array}$$
  (16) $$\begin{array}{l} \text{s}\times \text{t}\equiv \text{u}\left(\text{modr}\right) \end{array}$$
  (17) $$\begin{array}{l} \frac{\text{s}}{\text{t}}\equiv \text{u}\left(\text{modr}\right) \end{array}$$
3) *a, b* are positive numbers between 0 and *r-1*, and the curve is denoted *W*_*r*_(*s, t*).

The calculation process of the ECC algorithm is as follows:

1) The sender A selects an elliptic curve *W*_*r*_*(s,t)*, and selects a point *F* on the curve as the base point.
2) The sender A selects a private key *P*_2_ and calculates the public key *P*_1_ by Formula 18.
  (18) $$\begin{array}{l} {\text{P}}_{1}={\text{P}}_{2}\text{F} \end{array}$$
3) A sends the elliptic curve *W*_*r*_ *(s, t)* and the public key *P*_1_, point F to the recipient B.
4) After receiving the message, B sends the plaintext code to the point Q on the elliptic curve *W*_*r*_ *(s, t)* to generate a random integer *l*.
5) Receiver B calculates the point as Formula 19:
  (19) $$\begin{array}{l} {\text{u}}_{1}=\text{Q}+\text{l}{\text{P}}_{1},\;{\text{u}}_{2}=\text{lF} \end{array}$$
6) B sends the values of *U*_1_ and *U*_2_ to A.
7) A solve Formula 20:
  (20) $$\begin{array}{l} {\text{U}}_{1}-{\text{P}}_{2}{\text{U}}_{2}=\text{q}+\text{l}{\text{P}}_{2}\text{F}-{\text{P}}_{2}\text{lF}=\text{Q} \end{array}$$
8) A decodes Q to get the original plaintext.

The attacker can only get the elliptic curve *W*_*r*_*(s,t), F, P*_1_, *U*_1_, *U*_2_, but it is difficult to get Q because there is no private key *P*_2_.

## Experimental results of college students' physical fitness data monitoring based on the internet of things and blockchain

### Experiment objects and methods

#### Experiment objects

The research object of this paper is the physical fitness test data of students in the class of 2019 in Vocational and Technical College A from 2019 to 2021, and the survey objects are full-time and non-sports college students. Menstruating women with major organ lesions, systemic disabilities, or severe acute disease were excluded from the study.

The test data in this article are derived from the results of students' physical fitness test in the class of 2019. The instruments and test methods, test content, and scoring standards used in the test are strictly in accordance with the content and requirements stipulated in the “National Student Physical Health standard.”

Vocational and Technical College A has 2,202 students in the class of 2019, including 821 boys and 1,381 girls. The average age of students at school is 18.986 years old.

#### Research methods

Based on the needs of scientific research, a large number of domestic and foreign literature resources related to this research have been consulted, the progress was grasped in this field, and a scientific theoretical foundation and basic ideas for problem solving were laid for this research. At the same time, through the internet cnki, a lot of books and articles on student health care were searched, and a lot of literature related to this topic were also consulted. Theoretical and methodological basis were provided to the research of this article after analyzing, summarizing, and researching.

To ensure the correctness of the test data, a unified pretest technical training was carried out for all testers. This article mainly studied the physical test data of the 2019 class of students from 2019 to 2021. Based on the blockchain technology, the monitoring and research of the physical fitness data of college students was carried out. The test contents included : Lung capacity, standing long jump, sitting forward bend, 50-meter sprint, men's 1-kilometer run, women's 800-meter run, men's pull-ups, and women's one-min sit-ups.

At the same time, by using the logical analysis of synthesis, deduction, contrast, synthesis, etc., the conclusions of the mathematical statistics in this paper were summarized and analyzed, and a deeper research was carried out to analyze the current state of the physique, existing problems and their causes of the 2019 class of students of Vocational and Technical College A in 2019–2021, demonstrating relevant conclusions and propose corresponding countermeasures.

### Physical function indicators

Vital capacity refers to the amount of air that is exhaled to the best of its ability after a maximum inhalation. It represents the maximum functional activity of the lungs and is one of the important functional indicators reflecting the growth and development of body function. Vital capacity includes tidal volume, supplemental inspiratory volume, and supplemental expiratory volume. Vital capacity is the maximum ventilation volume for one breath, which can reflect the potential capacity of respiratory function to a certain extent. According to the national Student physical health standard, the excellent lung capacity of college students is 4,800 ml for boys and 3,300 ml for girls; the good level is 4,300 ml for boys and 3,000 ml for girls; the pass level is 3,100 ml for boys and 2,000 ml for girls. The vital capacity of the 2019 class of male and female students, at Vocational and Technical College A, was measured separately for 3 years. A total of 2,202 students were measured, including 821 males and 1,381 females. The results are shown in Table 1.

**Table 1:** Student vital capacity.

**Years**	**Boy**				**Girl**			
	**Vital capacity**	**Years**	**Difference in mean**	**Salience**	**Vital capacity**	**Years**	**Difference in mean**	**Salience**
2019	3,710.13	2020	−40.02	0.221	2424.01	2020	43.41	0.011
		2021	−30.97	0.341		2021	51.97	0.002
2020	3,750.02	2019	40.03	0.221	2379.97	2019	−43.41	0.011
		2021	8.91	0.791		2021	9.11	0.599
2021	3,739.96	2019	30.97	0.341	2369.95	2019	−51.97	0.002
		2020	−8.91	0.791		2020	−9.11	0.599

It can be seen from Table 1, the average lung capacity of boys from 2019 to 2021 was 3,710.13, 3,750.02, and 3,739.96 ml; the average lung capacity of girls from 2019 to 2021 was 2,424.01, 2,379.97, and 2,369.95 ml, respectively. These data show that boys' lung capacity score have increased in 2020 and decreased in 2021; girls' lung capacity score have showed a downward trend over the 3 years.

The analysis of variance was performed on the three-year lung capacity samples of 2019 students. The analysis of variance for the lung capacity of boys F was 0.833, and the significance was about 0.435, *p* > 0.05, which proved that there was no significant difference in the lung capacity of boys in the past 3 years. The variance analysis of girls' vital capacity F was 5.224, and the significance is about 0.005, *p* < 0.01, which proved that there was a significant difference in the vital capacity of girls in the past 3 years.

**Figure 4:**
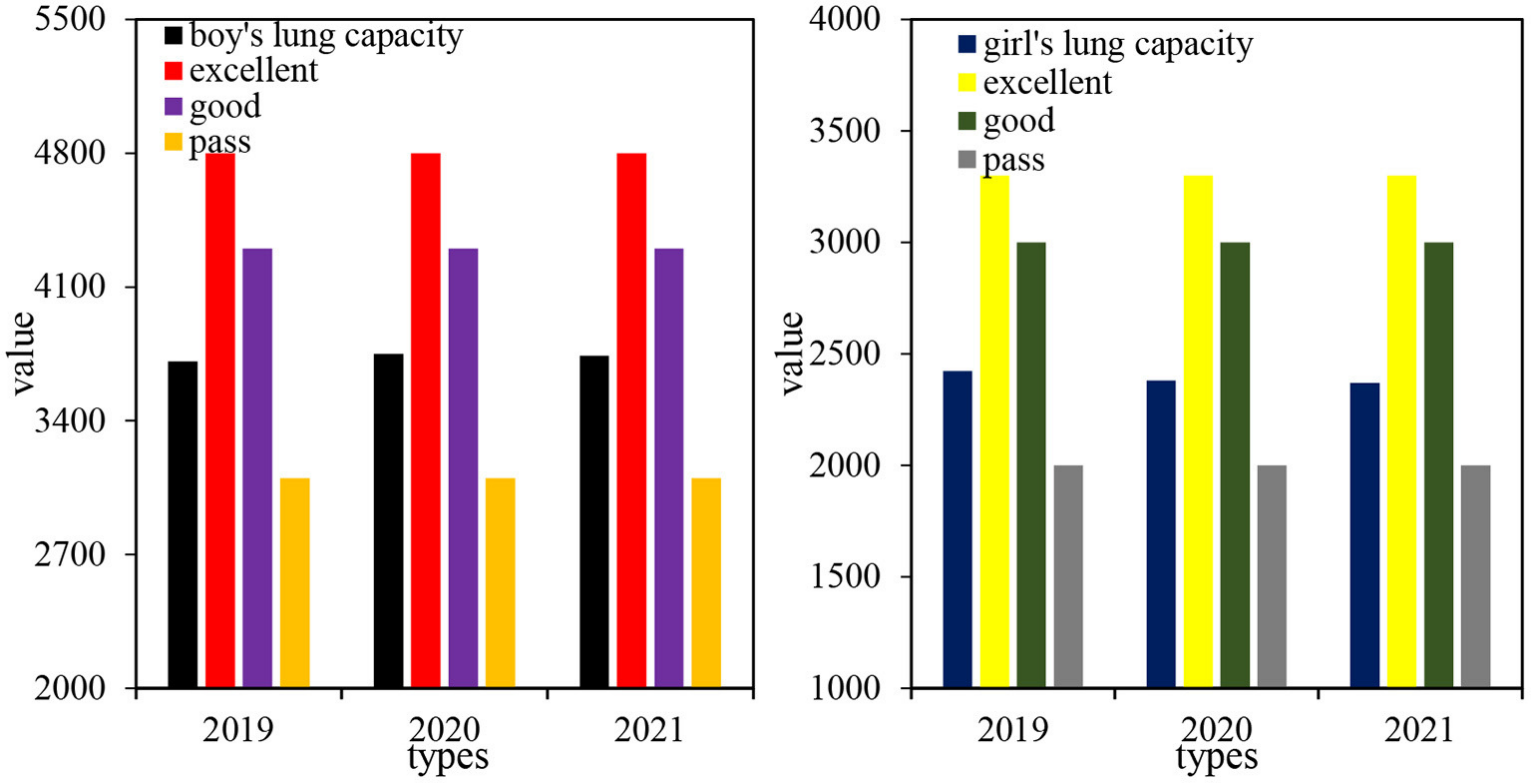
Comparison of average lung capacity of boys and girls.

It can be seen from Figure 4 that from 2019 to 2021, the lung capacity score of boys increased by 29.83 ml. The lung capacity score of boys increased in the second grade and decreased a little in the third grade. The standard deviation of boys' vital capacity had a large dispersion in 2021, and the boys' vital capacity levels in the 3 years were between the good and pass level. From 2019 to 2021, girls' lung capacity scores decreased by 54.06 ml, and girls' vital capacity scores decreased year by year for 3 years. The standard deviation of girls' vital capacity in 2020 was relatively large, and the girls' vital capacity level in 3 years was between the good and pass level.

### Physical fitness indicators

#### Standing long jump

Standing long jump refers to a long jump that starts from a standing position without a run-up, which reflects the level of explosive power of the lower limbs of the human body. According to the national student Physical Health standard, the outstanding standing long jump of college students is 263 cm for boys and 195 cm for girls; the good level is 248 cm for boys and 181 cm for girls; the pass level is 208 cm for boys and 151 cm for girls. The standing long jump of male and female students in class 2019 of Vocational and Technical College A was measured separately for 3 years. The results are shown in Table 2.

**Table 2:** Student standing long jump.

**Years**	**Boy**				**Girl**			
	**Standing long jump**	**Years**	**Difference in mean**	**Salience**	**Standing long jump**	**Years**	**Difference in mean**	**Salience**
2019	212.01	2020	0.598	0.509	151.02	2020	−1.09	0.061
		2021	−0.781	0.399		2021	−4.39	0
2020	210.97	2019	−0.598	0.509	151.98	2019	1.09	0.061
		2021	−1.401	0.141		2021	−3.31	0
2021	213.03	2019	0.781	0.399	154.02	2019	4.39	0
		2020	1.401	0.141		2020	3.31	0

It can be seen from Table 2 that the average standing long jump of boys from 2019 to 2021 was 212.01, 210.97, and 213.03 cm, respectively; the average standing long jump of girls from 2019 to 2021 was 151.02, 151.98, and 154.02 cm. These data show that the standing long jump performance of boys has decreased in 2020 and increased in 2021; the standing long jump performance of girls has showed an upward trend in the past 3 years.

A variance analysis was performed on the three-year standing long jump sample of the 2019 class of students. The male standing long jump analysis of variance F was 1.122, and the significance was about 0.326, *P* > 0.05, which proved that there was no significant difference in the male standing long jump in the past 3 years. The female standing long jump analysis of variance F was 30.350, *P* < 0.01, which proved that there was a significant difference in the standing long jump among girls in the past 3 years.

**Figure 5:**
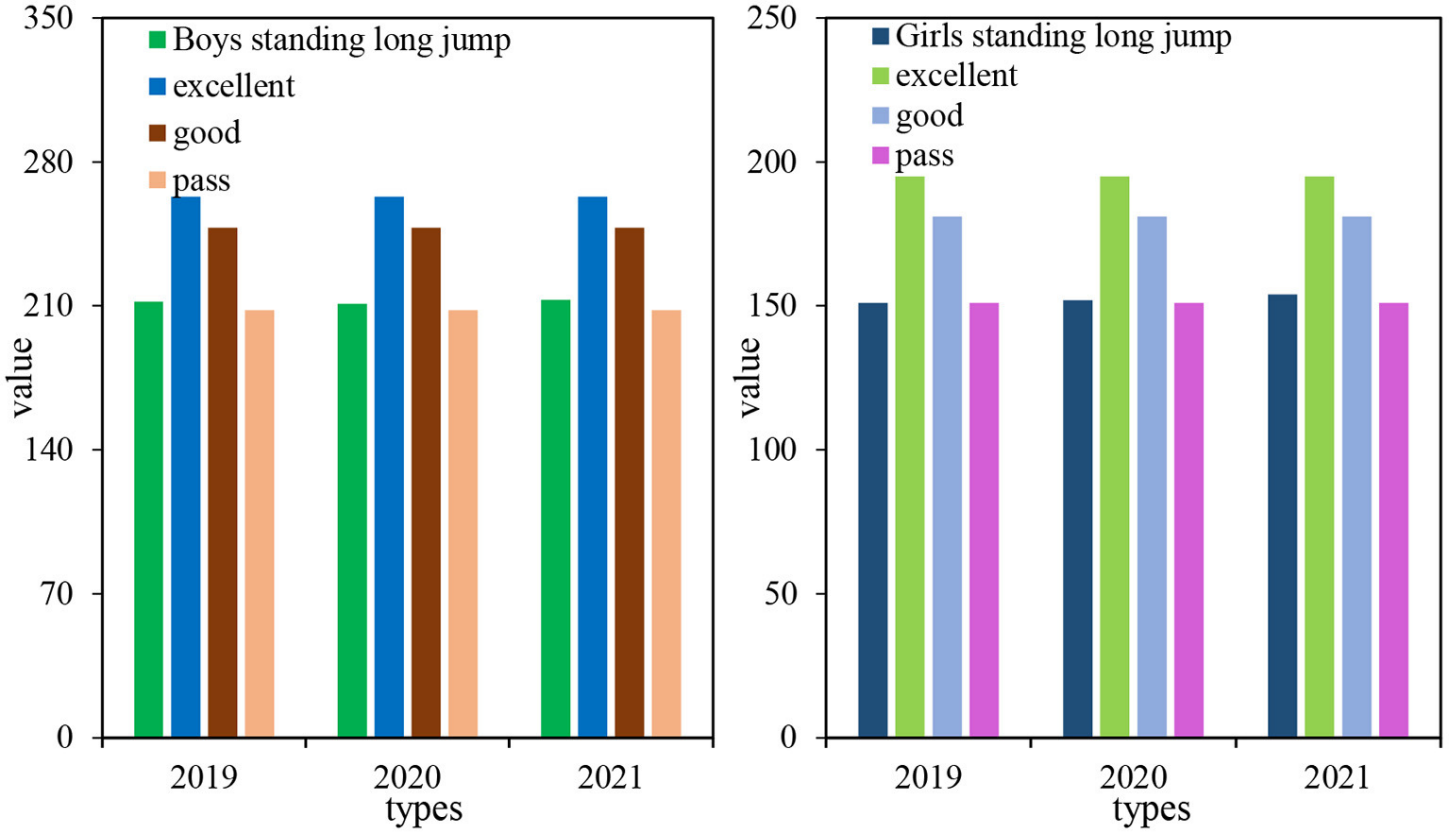
Comparison of the average values of standing long jump for boys and girls.

Figure 5 shows that the standing long jump performance of boys has increased by 1.02 cm from 2019 to 2021. The standing long jump performance of boys decreased a little in the second grade and increased in the third grade. The standard deviation of the standing long jump of boys in 2021 was relatively large, and the standing long jump level of boys in 3 years was between the good and pass level. From 2019 to 2021, the standing long jump performance of girls increased by 3 cm, and the standing long jump performance of girls increased slowly for 3 years. In 2019, the standard deviation of girls' standing long jump was relatively large, and the level of girls' standing long jump in 2019 was not up to standard, and in 2020 and 2021, it was between good and pass levels.

#### Sitting forward bend

Sitting forward bend is a measure of the maximum range of motion that can be achieved in joints such as the trunk, waist, and hip in a static state. It mainly reflects the extension and elasticity of the joints, ligaments, and muscles of these parts and the development level of the flexibility of the body. According to the national student physical health standard, the excellent level of sitting forward bending for college students is 21.3 cm for boys and 22.2 cm for girls; the good level is 17.7 cm for boys and 19.0 cm for girls; the pass level is 3.7 cm for boys and 6.0 cm for girls. The seated forward flexion of male and female students in class 2019 of Vocational and Technical College A was measured separately for 3 years, and the results are shown in Table 3.

**Table 3:** Students sit forward bend.

**Years**	**Boy**				**Girl**			
	**Standing long jump**	**Years**	**Difference in mean**	**Salience**	**Standing long jump**	**Years**	**Difference in mean**	**Salience**
2019	11.71	2020	0.621	0.069	11.49	2020	−0.869	0
		2021	−2.291	0		2021	−4.198	0
2020	11.11	2019	−0.621	0.069	12.41	2019	0.869	0
		2021	−2.951	0		2021	−3.401	0
2021	13.98	2019	2.291	0	15.81	2019	4.198	0
		2020	2.951	0		2020	3.401	0

It can be seen from the table that the average sitting body flexion of boys from 2019 to 2021 was 11.71, 11.11, and 13.98 cm, respectively; the average sitting forward flexion of girls from 2019 to 2021 was 11.49, 12.41, and 15.81 cm, respectively. These data show that boys' sitting front bending performance has decreased in 2020 and increased in 2021; girls' sitting front bending performance has shown an upward trend in the past 3 years.

The three-year sitting body forward flexion sample of 2019 students was analyzed by variance analysis. The variance analysis of male sitting body forward flexion F was 40.700, and the significance was about zero, *P* < 0.01, which proved that there was a significant difference in the male sitting forward flexion in the past 3 years. There is a significant difference in flexion. The analysis of variance of female sitting body forward flexion F was 201.382, the significance was about zero, *p* < 0.01, which proved that there was a significant difference in female sitting forward flexion in the past 3 years.

**Figure 6:**
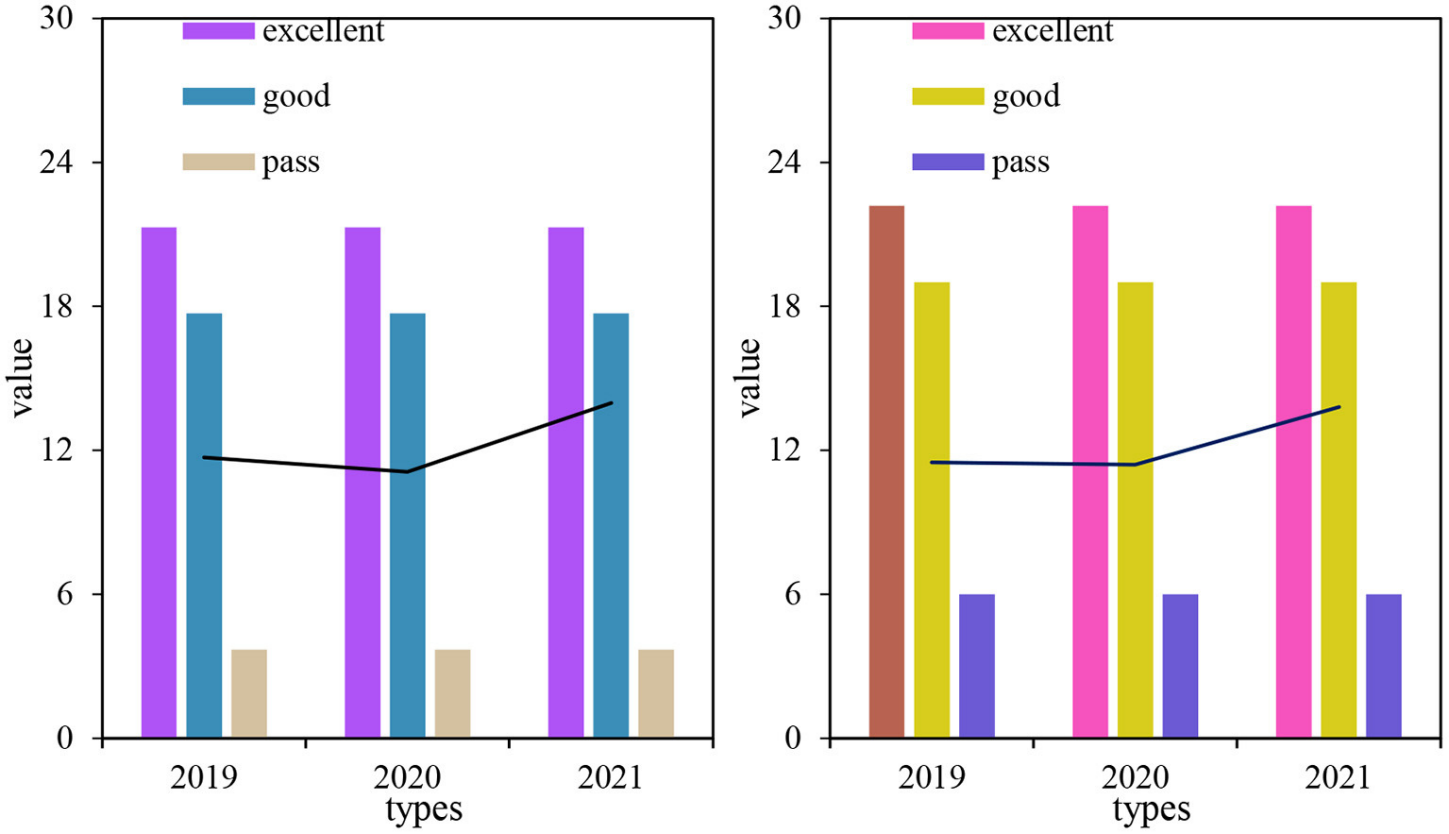
Comparison of the average values of male and female sitting forward flexion.

It can be seen from Figure 6 that from 2019 to 2021, boys' sitting forward flexion score increased by 2.27 cm, while boys' sitting forward flexion scores decreased a little in the second grade and improved in the third grade. In 2020, the standard deviation of males sitting forward flexion was relatively large, and the three-year male sitting forward bending level was between the good and pass level. From 2013 to 2015, the performance of girls' sitting body forward bending increased by 3.32 cm, and the girls' sitting body forward bending performance increased slowly for 3 years. In 2013, the standard deviation of female seated forward flexion was relatively large, and the level of female seated forward flexion for 3 years was between the good and pass level.

#### 50m dash

The 50-meter run is a sport that can reflect fast running and reaction ability. According to the national student physical health standard, the 50 m run for college students is 6.9 s for boys and 7.7s for girls at the excellent level; 7.1 s for boys and 8.3s for girls at the good level; 9.1 s for boys and 10.3 s for girls at the pass level. The 50 m running of male and female students in class 2019 of Vocational and Technical College A in 3 years was measured separately. The results are shown in Table 4.

**Table 4:** Student 50 m run.

**Years**	**Boy**				**Girl**			
	**50 m run**	**Years**	**Difference in mean**	**Salience**	**50 m run**	**Years**	**Difference in mean**	**Salience**
2019	8.21	2020	−0.089	0.019	10.69	2020	−0.069	0.079
		2021	0.239	0		2021	0.671	0
2020	8.31	2019	0.089	0.019	10.81	2019	0.069	0.079
		2021	0.341	0		2021	0.698	0
2021	8.01	2019	−0.239	0	9.98	2019	−0.671	0
		2020	−0.341	0		2020	−0.698	0

It can be seen from the table that the average 50 m run for boys in 2019-2021 is 8.21 s, 8.31 s, and 8.01 s, respectively; the average 50 m run for girls in 2019-2021 is 10.69 s, 10.81 s, and 9.98 s, respectively. These data show that male and female 50 m running performance decreased in 2020 and increased in 2021.

The three-year 50 m running sample of 2019 students was analyzed by variance analysis. The variance analysis F of the boys' 50 m running was 36.287, and the significance was about 0, *P* < 0.01, which proved that there was a significant difference in the boys' 50 m running in the past three years. The variance analysis f of the girls' 50 m running was 201.229, the significance is about 0, *p* < 0.01, which proved that there were significant differences in the girls' 50 m running in the past 3 years.

**Figure 7:**
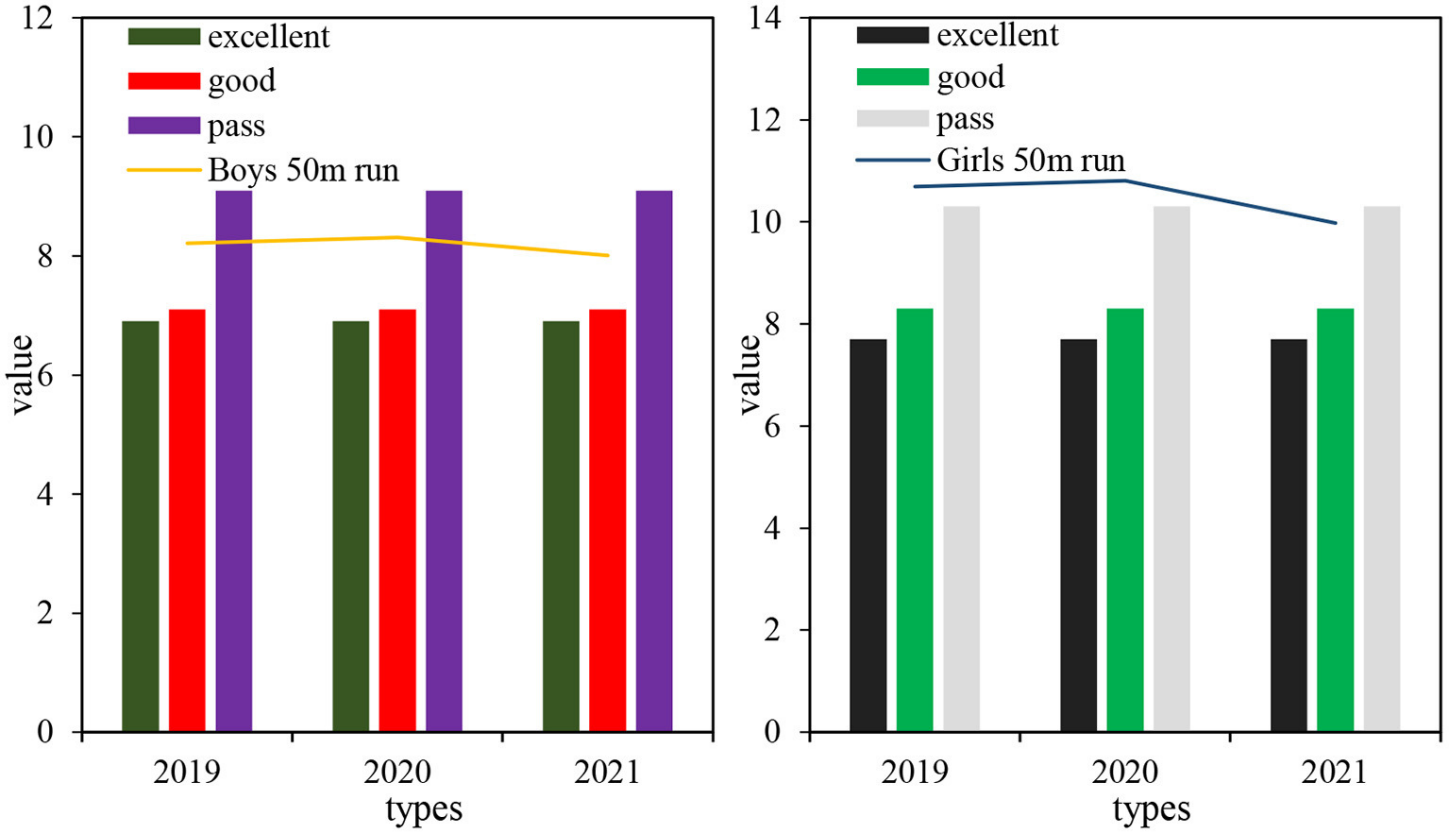
Comparison of the average of boys and girls in 50 m running.

As can be seen from Figure 7, the boys' 50 m running performance increased by 0.1 s from 2019 to 2020, the boys' 50 m running performance decreased a little in the second grade, and increased in the third grade. The standard deviation of boys' 50 m running in 2021 was relatively large, and the boys' 50 m running level in 3 years was between the good and pass level. From 2019 to 2021, the girls' 50 m running performance increased by 0.71 s. The girls' 50 m running performance decreased a little in the second grade and improved in the third grade. The standard deviation of girls' 50 m running in 2020 was relatively large, and the level of girls' 50 m running was in the unqualified and unqualified levels in 2019 and 2020, and was between the good and the passing level in 2021.

#### Men's 1,000 m run and women's 800 m run

Middle-distance running refers to middle-distance running and long-distance running. It belongs to the track and field sports with a distance of more than 800 meters. Middle-distance running is a comprehensive sport that requires speed and endurance. The human body needs to be able to run at a high speed for a long time. According to the national student physical Health standard, the 1,000 m running for college boys is 3.27 min at the excellent level, 3.42 min at the good level, and 4.32 min at the pass level; for girls, 3.30 min at the excellent level, 3.44 min at the good level, and 4.34 min at the pass level. The 1,000 m run for boys and the 800 m run for girls in the class of 2019 in Vocational and Technical College A were measured separately in 3 years. The results are shown in Table 5.

**Table 5:** The 1,000 m run for boys and 800 m run for girls in the class of 2019 in Vocational and Technical College A.

**Years**	**Boy**				**Girl**			
	**1000 m run**	**Years**	**Difference in mean**	**Salience**	**800 m run**	**Years**	**Difference in mean**	**Salience**
2019	4.21	2020	−0.521	0	4.09	2020	−0.319	0
		2021	−0.301	0		2021	−0.271	0
2020	4.71	2019	0.521	0	4.39	2019	0.319	0
		2021	0.219	0		2021	0.049	0
2021	4.39	2019	0.301	0	4.41	2019	0.271	0
		2020	0.219	0		2020	−0.049	0

It can be seen from the table that the average 1,000 m run for boys from 2019 to 2021 is 4.21, 4.71, and 4.39 min, respectively; the average of 800 m run for girls is 4.39 min, 4.39 min and 4.41 min respectively from 2019 to 2021. These data show that boys' 1000 m running performance and girls' 800 m running performance have decreased in 2020 and increased in 2021.

For the three-year 1,000 m running for boys and 800 m running for girls in 2019 students, the variance analysis was carried out. The variance analysis F of the boys' 1000 m running was 116.766, and the significance was about zero, *P* < 0.01, which proved that there was a significant difference in the boys' 1000 m running in the past 3 years. The analysis of variance (F) of the 800 m running for girls was 152.028, and the significance was about zero, *P* < 0.01, which proved that there were significant differences in the 800 m running for girls in the past 3 years.

**Figure 8:**
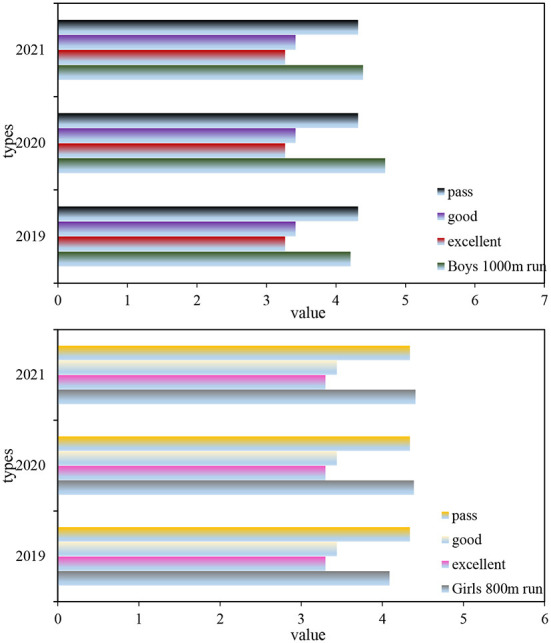
Comparison of the average value of boys' 1,000 m running and girls' 800 m running.

It can be seen from Figure 8 that the boys' 1,000 m running performance decreased by 0.18 min from 2019 to 2021, the boys' 1,000 m running performance decreased in the second grade, and increased a little in the third grade. The standard deviation of boys' 1,000 m running in 2020 was relatively large. The boys' 1,000 m running level was between good and passing levels in 2019, and was not up to standard in 2020 and 2021. From 2019 to 2021, the girls' 800 m running performance decreased by 0.32 min, the girls' 800 m running performance decreased in the second grade, and increased a little in the third grade. The standard deviation of girls' 800 m running in 2021 was relatively large. The girls' 800 m running level in 2019 was between good and passing levels and failed to meet the standard in 2020 and 2021.

### Male pull-ups and female sit-ups

The pull-up mainly reflects the development level of upper limb muscle strength and endurance. It is a dangling strength exercise that uses its own upper limb strength to overcome its own gravity. It is one of the important reference standards for measuring male physique. Pull-ups play an important role in the development of upper body suspension strength, shoulder girdle strength, and grip strength. It is a strength endurance event. Sit-ups mainly reflect the development level of waist and abdominal muscle strength and endurance. It can not only enhance the elasticity of the abdominal muscles, but also achieve the effect of protecting the back and improving body shape.

According to the national student physical Health standard, college boys have 17 pull-ups at the excellent level, 15 times at the good level, and 10 at the pass level; for girls, 52 times in sit-ups are at the excellent level, 46 times at the good level, and 26 times at the pass level. The boys' pull-ups and girls' sit-ups were measured separately for students of the class of 2019 in Vocational and Technical College A in the past 3 years. The results are shown in Table 6.

**Table 6:** Male pull-up and female sit-up.

**Years**	**Boy**				**Girl**			
	**Pull-ups**	**Years**	**Difference in mean**	**Salience**	**Sit-ups**	**Years**	**Difference in mean**	**Salience**
2019	8.02	2020	2.729	0	25.41	2020	4.31	0
		2021	1.608	0		2021	0.298	0
2020	5.21	2019	−2.729	0	21.09	2019	−4.31	0
		2021	−1.109	0		2021	−4.01	0
2021	6.29	2019	−2.729	0	25.11	2019	0.298	0
		2020	1.109	0		2020	4.01	0

It can be seen from the table that the average pull-up times for boys from 2019 to 2021 are 8.02 times, 5.21 and 6.29 times, respectively; the average number of sit-ups for girls from 2019 to 2021 is 25.41, 21.09, and 25.11 times, respectively. These data show that both boys' pull-up performance and girls' sit-up performance decreased in 2020 and increased in 2021.

Analysis of variance was performed on the three-year boys' pull-ups and girls' sit-ups samples of the 2019 class of students. The analysis of variance F for boys' pull-ups was 76.222, and the significance was about 0, p <0.01, which proved that these 3 years there were significant differences in male pull-ups. The analysis of variance for girls' sit-ups was 89.187, the significance was about zero, *p* < 0.01, which proved that there were significant differences in girls' sit-ups in the past 3 years.

**Figure 9:**
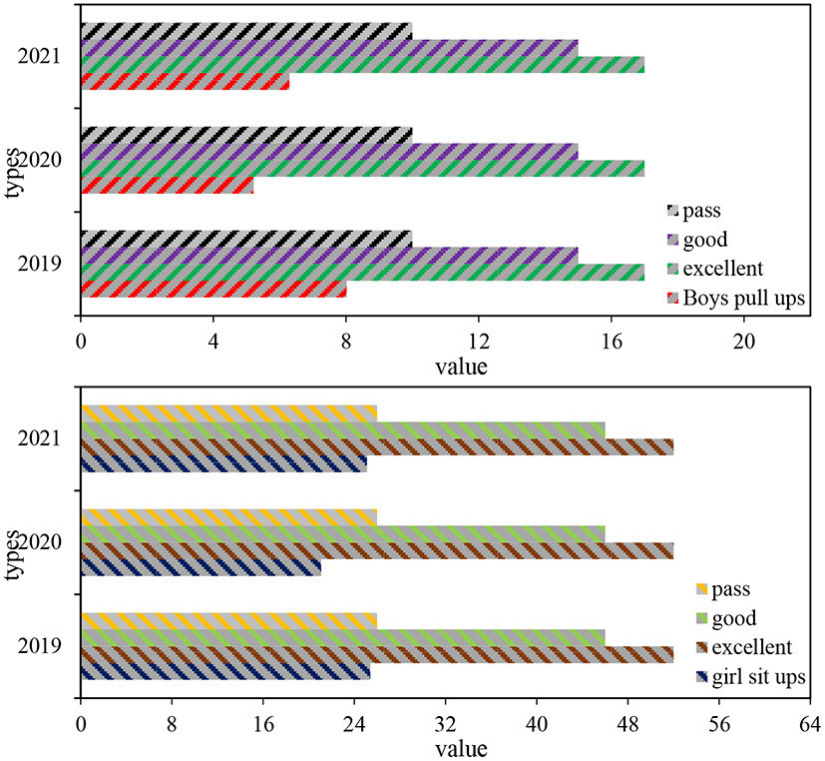
Average comparison of male pull -ups and female sit-ups.

As can be seen from Figure 9, the chin-up performance of boys decreased by 1.73 times from 2019 to 2021, the chin-up performance of boys decreased in the second grade, and increased a little in the third grade. The standard deviation of boys' pull-ups in 2019 was relatively large, and the level of male pull-ups in 2013, 2014, and 2015 were all unqualified and not up to standard. Girls' sit-up scores decreased by 0.3 from 2019 to 2021. From 2019 to 2021, girls' sit-up scores decreased by 0.3 times, and the girls' sit-ups scores decreased in the second grade and increased a little in the third grade. The standard deviation of girls' sit-ups in 2021 was relatively large, and the level of girls' sit-ups in 2019, 2020, and 2021 were unqualified and not up to the standard.

## Discussion

In the past 3 years, boys' pull-ups and girls' sit-ups have decreased in 2020 and increased in 2021. There were significant differences in boys' pull-ups and female sit-ups in the past 3 years. The gap in the level of pull-ups for boys is relatively large in 2019; the gap in the level of sit-ups for girls is relatively large in 2021. The level of pull-ups for boys and sit-ups for girls in 2019, 2020, and 2021 were all unsatisfactory. The results showed that the level of pull-ups for boys and sit-ups for girls decreased significantly in the second grade and improved in the third grade. It showed that the second grade boys did not often participate in upper body strength exercises; the second grade girls did not often participate in abdominal strength exercises. The level of pull-ups for boys and sit-ups for girls was very low, and they have been in a state of failing to meet the standards for 3 years. Mainly, because boys lack upper limb strength, girls lack abdominal strength. Boys lack upper limb muscle strength and endurance exercise, and girls lack waist and abdomen muscle strength and endurance exercise. In this regard, schools should comprehensively promote quality education, vigorously publicize the idea of “health first,” and ensure that college students have time to participate in physical exercise. Stabilize the school's existing teaching mode, strengthen the cultivation of college students' awareness of physical exercise, and mobilize teachers and students in the whole school to pay attention to college students' physical health education.

## Conclusion

To test the physical fitness of college students, this paper conducted a physical fitness test on the 2019 college students of Vocational and Technical College A based on block technology. During the 3 years in school, the indicators of the students generally fell. The level of lung capacity in boys did not change significantly, while in girls it showed a downward trend. There was no significant difference in the performance of boys standing long jumpers, but the performance of girls gradually increased. In the second grade, the level of forward flexion of the male sitting body decreased, but increased significantly in the third grade; the female showed an upward trend. The 50 m running level decreased in the second grade, but improved significantly in the third grade. In the second grade, the level of boys' 1,000 m running and girls' 800 m running dropped significantly, but it improved in the third grade. The level of pull-ups for boys and sit-ups for girls decreased significantly in the second grade, but improved in the third grade. To strengthen the physical quality of the students, the investment should be further increased rationally, the sports venues on campus will be planned, and sports facilities and equipment as much as possible will be provided. At the same time, students should be encouraged and supported to set up various sports associations and societies, and physical education teachers should be arranged to provide scientific guidance. However, due to the limitation of time and technology, the specific application of block technology still needs to be studied. Further research and discussion in the follow-up will be conducted.

## Data availability statement

The original contributions presented in the study are included in the article/supplementary material, further inquiries can be directed to the corresponding author.

## Ethics statement

Ethical review and approval was not required for the study on human participants in accordance with the Local Legislation and Institutional requirements. Written informed consent from the [patients/participants legal guardian/next of kin] was not required to participate in this study in accordance with the national legislation and the institutional requirements.

## Author contributions

YS designed the experiments, collected data for experimental comparison usage, ran the experiments for the performance study, and wrote the first draft of the paper. LW critically reviewed the method and contributed to structuring the paper. All authors contributed to the article and approved the submitted version.

## Conflict of interest

The authors declare that the research was conducted in the absence of any commercial or financial relationships that could be construed as a potential conflict of interest.

## Publisher's note

All claims expressed in this article are solely those of the authors and do not necessarily represent those of their affiliated organizations, or those of the publisher, the editors and the reviewers. Any product that may be evaluated in this article, or claim that may be made by its manufacturer, is not guaranteed or endorsed by the publisher.
